# Migration analysis of a metaphyseal anchored short-stem hip prosthesis: EBRA-FCA evaluation of 80 implants with a minimum follow-up time of 2 years

**DOI:** 10.3109/17453674.2012.712891

**Published:** 2012-08-25

**Authors:** Florian Schmidutz, Thomas Graf, Farhad Mazoochian, Andreas Fottner, Andrea Bauer-Melnyk, Volkmar Jansson

**Affiliations:** ^1^Departments of Orthopedic Surgery; ^2^Radiology, University of Munich (LMU), Campus Grosshadern, Munich, Germany

## Abstract

**Background and purpose:**

Metaphyseal anchored short-stem hip implants were designed to improve load transmission and preserve femoral bone stock. Until now, only few outcome data have been available and migration studies are one of the few ways of obtaining data that are predictive of implant survival. We therefore evaluated a metaphyseal anchored short-stem hip implant by Ein Bild Roentgen Analyse femoral component analysis (EBRA-FCA).

**Patients and methods:**

First, the EBRA-FCA method was validated for the short-stem hip implant. Then 80 of the first 100 consecutive implants were evaluated after at least 2 years. Clinical assessment was performed using the WOMAC and the UCLA score.

**Results:**

After 2.7 (2.0–4.2), years none of the implants had been revised and by that time the stems had subsided by a mean of 0.7 mm (SD 1.8) (95% CI: 0.3–1.1). Of the 80 implants, 78 were stable after 2 years, with 74 being primary stable and 4 showing secondary stabilization after initial subsidence. Continuous migration was seen in only 2 patients. The clinical outcome showed good results with a mean WOMAC of 11 (SD 13) and a mean UCLA score of 7.3 (SD 2.0). [OK?]

**Interpretation:**

The metaphyseal anchored short-stem hip implant showed good functional results and a high degree of stability after 2 years. The outcome is comparable to that of clinically proven conventional hip implants and if the results are confirmed by long-term studies, short-stem hip arthroplasty might be an alternative for young patients requiring hip replacement.

The number of young patients with total hip arthroplasty is steadily increasing. However, it is well known that young patients face a higher risk of implant failure (Garellick et al. 2010), which makes it very likely that they will have at least 1 revision during their lifetime. At revision, loss of bone stock is common and makes it difficult to anchor the revision implant. The use of bone conserving implants may facilitate revision.

Short-stem hip implants are shorter than standard stems and the first designs, the Mayo and the Pipino short stem, were already introduced several years ago (Pipino and Calderale 1987, Morrey 1989). Due to the encouraging results (Morrey et al. 2000, Briem et al. 2010), various new short-stem implants have recently been developed and are being implanted in increasing numbers. However, barely any data exist about the long-term outcomes and they will not be available in the near future. For this reason, it is important to evaluate these implants shortly after their introduction to obtain data that may help predict their survival.

Initial stability and early migration of implants have been shown to predict long-term survival (Freeman and Plante-Bordeneuve 1994, Krismer et al. 1999). Several methods have been published, with radiostereometric analysis (RSA) and Ein Bild Roentgen Analyse femoral component analysis (EBRA-FCA) proving to be the most dependable ones (Selvik 1989, Krismer et al. 1995). With an accuracy of 0.2 mm, RSA is the most precise one, but it requires prospective planning, implantation of tantalum markers, and special stereoradiographs (Karrholm et al. 1997). In contrast, EBRA-FCA is a non-invasive method, which allows retrospective analysis of routinely taken AP radiographs. It still offers high precision, with a specificity of 100% and a sensitivity of 78% for detecting an implant migration of 1 mm (Biedermann et al. 1999). The present study was designed to evaluate the early migration of a short-stem implant with EBRA-FCA, and also to evaluate the functional outcome. Before being used, the EBRA-FCA method was first validated in vitro for the short-stem implant.

## Patients and methods

### Patients

The study cohort comprised the first 100 consecutively implanted short-stem hip arthroplasties at our orthopedic department (from May 2006 through November 2008). From this cohort, 82 implants (74 patients) could be evaluated. 10 patients were living abroad or had moved, and 8 refused to come to the clinic for reasons not related to the implant. The final study cohort comprised 43 males and 31 females, with a mean age of 55 (SD 12) years and a mean BMI of 27 (SD 4). Reasons for hip replacement were osteoarthritis (n = 34), dysplasia (n = 24), avascular necrosis of the femoral head (AVN) (n = 20), posttraumatic osteoarthritis (n = 1), and rheumatoid arthritis (n = 3). The mean follow-up time was 2.7 (2.0–4.2) years.

The study protocol was approved by the Ethics Committee of the university (No. 054-10). All patients were informed about the study and signed an informed consent form.

### Implants and surgery

All patients received the same short-stem hip implant (Metha; B. Braun Aesculap, Tuttlingen, Germany). The implant size ranges from 0 to 7, available as a monoblock (neck-shaft angle (CCD) 130°, 135°) or as a modular implant with cone adapters (130°, 135°, and 140°, all with 7.5° antetorsion, 7.5° retrotorsion, or 0°). Monoblock implants were used as follows: 130° (n = 3) and 135° (n = 3). Modular implants were used as follows:130°/0° (n = 27), 130°/7.5° ante (n = 4), 130°/7.5° retro (n = 2), 135°/0° (n = 32), 135°/7.5° ante (n = 8), 135°/7.5° retro (n = 1), 140°/0° (n = 1), and 140°/7.5° retro (n = 1).

For the acetabular component, either a threaded cup or a press-fit cup was used (Screwring SC or Plasmacup SC; B. Braun Aesculap, Tuttlingen, Germany). Surgery was performed by 3 senior surgeons (AF, FM, and VJ) with a minimally invasive anterolateral Harding approach in supine position. All implants were templated before surgery and evaluated intraoperatively by fluoroscopy. Weight bearing was allowed to half the body weight for the first 2 weeks, followed by a rapid increase to full weight bearing.

### Clinical study protocol

All patients had an AP radiograph postoperatively, and they were usually followed up at 3, 6, and 12 months, and then by annual assessment. Patients included in this study were required to have their implant longer than 24 months and to have at least 3 radiographs accepted by the EBRA-FCA software: 1 postoperatively, 1 within 2 years, and 1 at the final follow-up.

Clinical assessment at the last follow-up included the Western Ontario and McMaster Universities arthritis index (WOMAC), and the UCLA activity score. Radiographic evaluation included measurement of the CCD angle and the cup inclination. The size of the femoral cavity was evaluated, according to Noble et al. (1988), by measuring the endosteal distance at the midpoint of the lesser trochanter (point 1) and the endosteal distance 10 cm below (point 2).

### In vitro validation of EBRA-FCA for short-stem implants

**Figure 1.**
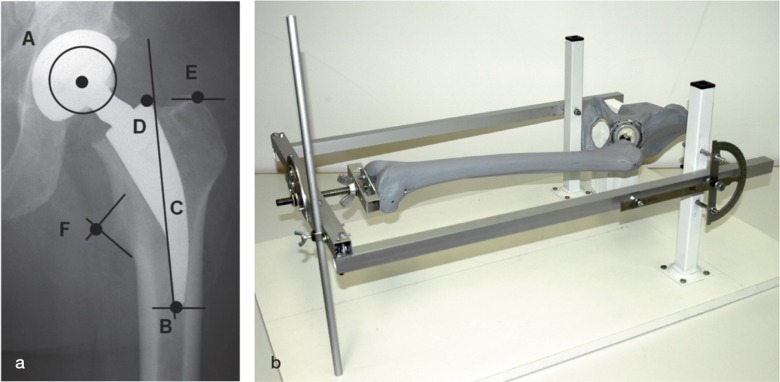
a. Reference points for EBRA-FCA analysis. A: center point of head; B: tip of stem; C: stem reference line; D: implant shoulder; E: greater trochanter; F: lesser trochanter. b. Experimental set-up for the in vitro evaluation.

Short-stem implants have a curved shape, which made it necessary for the author of the EBRA method to adapt the software and place the central axis as a tangent to the medial side of the implant (Figure 1A). Due to the different shape and the changed stem axis, EBRA-FCA was validated for the short-stem implant.

Validation was performed similarly to Ilchmann et al. (2006), who assessed a conventional stem in an in vitro model by a controlled-rotation and controlled-migration study (Figure 1B). The model was constructed by implanting a short-stem prosthesis (Metha, modular stem, size 2, 135°, neutral) into a left medium-sized Sawbone femur (model 1121-69; Sawbones Pacific Research Laboratories, Vashon, WA), and an acetabular cup (Screwring SC, size 52 mm) into a left medium-sized hemipelvis (model 1305-9; Sawbones). According to recent studies, the model allows a controlled flexion/extension, and a simultaneous external/internal rotation of the femur in 1° steps (Beaule et al. 2005, Ilchmann et al. 2006). Radiographs were acquired with the same settings and digital X-ray machine as for the patients, and calibration of the radiographs was performed using the implant head and an enclosed grid.

### Controlled-rotation study

A worst-case study was performed to evaluate possible rotational deviations of the femur that might occur during radiographic acquisition. According to recent studies, the evaluation was carried out at –5° and +20° of flexion, and at –10° and +50° of rotation (Beaule et al. 2005, Ilchmann et al. 2006). At 5 different time points, a series of radiographs at each position was taken and compared to the neutral position. Any difference between the neutral and the rotated position was taken to represent a rotation error.

### Controlled-migration study

To determine the measurement accuracy of an implant migration, a controlled implant subsidence was simulated. The implant was shifted down the femur from 0 to 6 mm. This was done in 1-mm steps with an integrated vernier calliper (Holex, Hoffmann Group, Nuremberg, Germany) firmly fixed at the implant and at the distal femur, allowing a precision of 0.05 mm. A series of radiographs from every single shift was taken at the 5 different time points. Any difference between the adjusted position and the measured position was taken to represent a measurement error.

### Migration analysis with EBRA-FCA

Migration analysis for the in vitro validation and for the implanted prosthesis was performed by the modified EBRA-FCA software (Institute for Basic Engineering Sciences, University of Innsbruck, Austria). The magnitude of stem migration is presented as mean (SD) with 95% confidence interval (CI).

### Statistics

To account for bilateral hips, we used a linear mixed model with a fixed intercept and a random effect for each patient, assuming a compound-symmetry covariance pattern for the repeated implantations (bilateral hip arthroplasty). The choice of the covariance pattern was supported by the Akaike information criterion. Statistical analysis was performed with SPSS 19.0. Graphs were created with SigmaPlot 8.02 (Systat Software GmbH, Erkrath, Germany).

## Results

### Clinical results

At the last postoperative follow-up (mean 2.7 (2.0–4.2) years), none of the stems or acetabular cups had been revised. At the last follow-up, the mean WOMAC was 11 (SD 13) (CI: 8–14), with 2 (SD 1) in the stiffness section, 2 (SD 3) in the pain section, and 8 (SD 10) in the function section. The activity level was rated on the UCLA scale, with a mean score of 7 (SD 2) (CI: 6.9–7.8).

The median size of the acetabular component was 54 (48–58) mm for men and 48 (44–52) mm for women. The median size of the stem was 4 (1–6) for men and 1 (0–4) for women. Radiographic evaluation showed a mean cup inclination of 45° (SD 6) and a mean CCD angle of 137° (SD 6). The mean endosteal diameter at the midpoint of the lesser trochanter (point 1) was 28 mm (SD 3.8) (range: 19.2–40), and 12 mm (SD 2.1) (range: 8.2–18) 10 cm below (point 2). The endosteal distances with respect to the implant size are given in Table 1.

**Table 1.** Size of the femur relative to the size of the non-migrated stems (mean (range))

Stem size	Point 1 ^a^, mm	Point 2 ^b^, mm
0	22 (19–25)	10 (9–11)
1	24 (20–27)	11 (9–16)
2	27 (24–29)	11 (10–12)
3	28 (23–32)	13 (9–16)
4	29 (22–32)	13 (11–15)
5	31 (26–40)	14 (11–18)
6	36 (35–37)	14 (12–15)

**^a^** Midline lesser trochanter (endosteal distance).

**^b^** 10 cm below the midline of the lesser trochanter (endosteal distance).

## In vitro validation results of EBRA-FCA for short stems

*Rotation error.* Compared to the neutral position, a flexion of 20° gave a maximum error of –1.5 mm with a mean of –1.5 mm (SD 0.1) (CI: –1.5 to –1.4). An extension of 5° gave a maximum error of +0.9 mm with a mean of +0.8 mm (SD 0.1) (CI: 0.8–0.9). External rotation was conducted at different angles, with 50°, 30°, and 20° being rejected by the software due to detected inaccuracy. The maximum accepted external rotation was 10°, which gave a maximum error of +0.2 mm with a mean of +0.1 mm (SD 0.1) (CI: 0–0.2). Internal rotation with 10° gave a maximum error of –0.2 mm with a mean of –0.1 mm (SD 0.1) (CI: –0.2 to 0) (Table 2).

**Table 2.** Validation of the EBRA-FCA method for the short stem and conventional stem. Values are mm

	SHA ^a^	THA ^b^
*Rotation*		
Flexion/extension 20°/5° and 16°/5° ^a^	–1.5 / +0.9	± 1.4
Internal/external rotation 10°/10° and 5°/5°	–0.2 / +0.2	± 0.7
*Migration*		
Controlled migration 0–6 and 0–7 mm ^a^	–0.5 / +0.4	± 0.5

**^a^** Short-stem hip arthroplasty, present study.

**^b^** Total hip arthroplasty (Ilchmann et al. 2006).

*Measurement precision.* Evaluation of the 5 controlled-migration series, running from the neutral position to 6 mm, resulted in a measured subsidence between 5.9 to 6.3 mm, indicating an error ranging from –0.1 mm to +0.3 mm with a mean of 0.0 mm (SD 0.2) (CI: –0.2 to 0.2). Analysis of each single step (1 mm) for all series gave an error ranging from –0.5 mm to +0.4 mm with a mean of 0.1 mm (SD 0.2) (CI: 0.0 to 0.1) (Table 2).

## Migration analysis

From the 82 implants, 2 series of radiographs were rejected by the EBRA-FCA software. The remaining 80 implants (73 patients) were analyzed with a mean of 4.5 (SD 1.8) (range 3–9) radiographs for each implant. The numbers of radiographs at defined time points were accepted and measured by EBRA-FCA as follows: n = 80 postoperatively, n = 80 at 3 months, n = 71 at 1 year, n = 68 at 2 years, n = 23 at 3 years, and n = 4 at 4 years.

**Figure 2.**
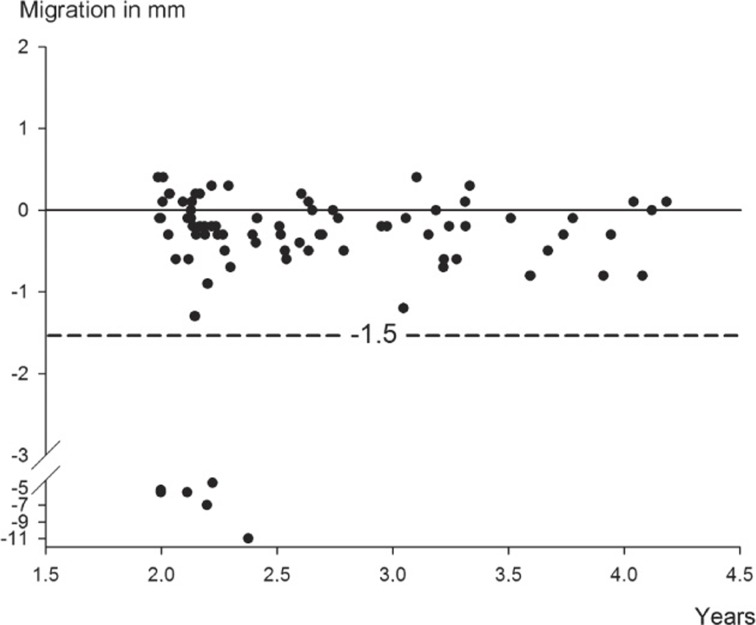
Subsidence of the short-stem implants at the final follow-up. The dashed line represents the critical threshold of –1.5 mm within the first 2 years (Krismer et al. 1999).

In the total study cohort, the mean vertical migration of the short-stem implants was –0.7 mm (SD 1.8) (CI: –1.1 to –0.3) after a mean of 2.7 (2.0–4.2) years. The implants were also assigned to 4 different patterns of migration (A–D) (Krismer et al. 1999). According to this classification, 74 implants were assigned to pattern D (primary stable) with a mean migration of –0.2 mm (SD 0.35) (CI: –0.3 to –0.1) (Figure 2). 6 implants (5 patients) showed early migration. 4 of the implants were assigned to pattern B, as they got secondary stable, and 2 of the implants were assigned to pattern A, as they showed a continuous subsidence (Figure 2, and Table 3).

**Table 3.** Data on the 6 migrated stems (5 patients)

A	B	C	D	E	F	G	H	I
1 - left	5.5	B	M	2	30/12	AVN		4 (1/1/3)
2 - right	5.2	B	M	2	31/11	AVN		4 (1/1/3)
3	7.0	B	M	4	28/13	Osteoarthritis	Parkinson	3 (0/0/3)
4	4.4	B	F	2	25/9	Dysplasia		0 (0/0/3)
5	11.1	A	M	2	30/14	AVN	Chronic alcohol abuse	0 (0/0/0)
6	5.5	A	M	2	32/15	AVN	High-dose steroids	20 (3/3/14)

AStem BMigration, mm CMigration pattern DSex EImplant Size FFemur size at point 1 / point 2, mm GDiagnosis HSecondary diagnosis IWOMAC: total WOMAC score (pain/stiffness/function).

From the 4 implants with pattern B, 3 migrated within the first 2 months and then stabilized. For the fourth implant, no definite time point within the first 2 years could be determined, as no sufficient radiographs were available in between. However, the implant did not migrate further after 2 years and showed complete osseous integration.

Of the 6 migrated stems, 4 (in 3 patients) were implanted due to AVN—1 due to osteoarthritis and 1 due to dysplasia. Both patients with continuous stem migration were diagnosed with AVN, 1 receiving high-dose steroid therapy due to kidney transplant rejection and 1 with severe chronic alcohol abuse (Table 3). From the 6 migrated implants, 4 stems (3 patients) showed a mismatch between the femur and the stem size. All 3 patients were male and received an implant size 2, but showed a larger medullary diameter of the femur than the other patients who received an implant size 2 (Tables 1 and 3).

## Discussion

Although only long-term results in hip arthroplasty should be considered valid, initial evaluation is necessary to identify undesirable results (Baad-Hansen et al. 2011). Early migration analysis allows prediction of implant survival (Krismer et al. 1999, Kroell et al. 2009), which is why we evaluated a metaphyseal anchored short-stem hip implant by EBRA-FCA.

EBRA-FCA is a reliable and accurate method (Biedermann et al. 1999, Kroell et al. 2009), but it has mainly been used for conventional total hip arthroplasty (THA) (Radl et al. 2005, Ince et al. 2006). Thus, we first evaluated the method for the short stem as previously described (Beaule et al. 2005, Ilchmann et al. 2006). The results obtained were similar to those for conventional THA (Ilchmann et al. 2006), so EBRA-FCA was considered to be a valid method for short-stem implants as well.

After 2.7 years, the mean subsidence was –0.7 mm, which is clearly below the reported critical threshold of –1.5 mm for the first 2 years (Krismer et al. 1999). This indicates a good stability and osseous integration for this short-stem implant and agrees with a biomechanical study that demonstrated a good primary stability (Fottner et al. 2009). As for the CFP short-stem implant, an RSA study reported a good stability after 2 years (Briem et al. 2010). The amount of migration is also comparable to that in migration studies of conventional cementless THA stems. For the Corail stem, a subsidence of –0.6 mm after 2 years has been reported (Campbell et al. 2011). For the CLS stem, a subsidence of –1.2 mm after 2 years (Strom et al. 2007) and –1.7 mm after 5 years (Wolf et al. 2010) has been reported. Both stem designs are known to have good long-term survival (Aldinger et al. 2003, Vidalain 2011).

As it is not only the average migration that is important, we assigned the implants to 4 different patterns of migration (A–D) (Krismer et al. 1999). 74 out of 80 short-stem implants were primary stable (pattern D) and none of them showed late-onset migration (pattern C). This is similar to the Mayo short stem, where Morrey et al. (2000) reported no subsidence in 88% of the cases after 6 years, while 5% showed less and 7% showed more than –2 mm of subsidence. For conventional THA stems, an EBRA-FCA study found less than –1 mm of migration in 71% of the implants (113 of 158) after 2 years (Krismer et al. 1999), compared to 72 of 80 implants in our study.

6 stems migrated more than –1.5 mm within the first 2 years, and 2 of them showed a continuous migration (pattern A) and were at risk of early failure. The remaining 4 implants showed an initial migration but then stabilized (pattern B).

A retrospective EBRA-FCA study with a 10-year follow-up showed that initial migration with secondary stabilization can succeed, and is not necessarily followed by implant failure (Krismer et al. 1999). Similar to our results, most of the recent RSA studies evaluating cementless THA stems have also found an initial subsidence, with a successful secondary stabilization within the first months (Thien et al. 2007, Strom et al. 2007, Wolf et al. 2010, Boe et al. 2011, Campbell et al. 2011). For 2 tapered THA designs, Boe et al. (2011) reported a mean subsidence of –0.3 mm within the first 3 months, followed by consistent stabilization. Campbell et al. (2011) reported an initial subsidence of up to –3.7 mm in 38% of the THA implants. Also, for the CLS stem, Wolf et al. (2010) reported an initial subsidence up to –6.7 mm in 16% of the stems within the first year. Success of secondary stabilization can be explained by the coating (Overgaard et al. 1997). However, stabilization of the short stem can also be explained by the curved and tapered shape of the stem design, which leads to a wedging in the proximal part of the femur, as described for the Mayo short stem (Morrey et al. 2000).

Only limited data are available regarding the cause of initial migration. Campbell et al. (2011) discussed the amount of impaction during stem insertion and the quality of the bone surrounding the implant as a possible reason for this initial subsidence. In the present study, 4 of the 6 stems that migrated were implanted due to AVN, which might have compromised bone quality in the metaphyseal region. These patients were all male and had been provided with a stem size 2, whereas males collectively had a median stem size of 4 (1–6). They also showed a larger femoral diameter than those patients with an implant size 2 and no subsidence. This combination of reduced bone quality and a mismatch between the femur and implant might have increased the risk of initial subsidence. Although this assumption is supported by a biomechanical study, which demonstrated that short-stem implant failure occurs with poor bone quality or suboptimal implantation (Westphal et al. 2006), further studies are required.

Functional results in our patients were good, with a mean WOMAC of 11. Similarly, good results were reported for the CFP short-stem implant with a HHS of 93 after 2 years and a HHS of 96 after 7 years (Rohrl et al. 2006, Briem et al. 2010). For the Mayo short-stem implant, a HHS of 93 was reported after 6.4 years (Morrey et al. 2000). We did not observe stress-shielding in terms of a sclerotic reaction at the distal tip of the stem on the radiographs, although a proportionately higher load has been described in a biomechanical study and in a finite element analysis (Speirs et al. 2007, Fottner et al. 2009). The risk of femur fracture during implantation is a concern (Jakubowitz et al. 2009), but this did not occur in our patients.

Our study has some limitations. Although EBRA-FCA is an accurate method, it mainly addresses the initial stability and does not take account of all loosening mechanisms, e.g. failure due to wear. Furthermore, the study was performed retrospectively, thus preoperative WOMAC had not been obtained and some patients could not be followed up. Still, we were able to analyze 80 of the first 100 implants, which is comparable to previous migration studies with an evaluation rate of 66–85% (Krismer et al. 1999, Rohrl et al. 2006, Buratti et al. 2009).

In summary, the early evaluation of the metaphyseally anchored short-stem implant revealed good clinical results and showed good stability comparable to that of clinically proven cementless THA stems. Migration was mainly seen in patients with avascular necrosis of the hip and with a possible mismatch between stem and femur size. After 2 years, 78 of 80 implants were stable and—if those results are confirmed by long-term studies—short-stem arthroplasty might be an alternative for young patients requiring hip replacement.
